# The TMAO Metabolic Axis in Vascular Disease: A Position Paper on Redox Mechanisms and Priorities for Clinical Translation

**DOI:** 10.3390/antiox15091109

**Published:** 2026-09-02

**Authors:** Francesca Miceli, Eugenio Caradonna, Claudia Panzano, Wassim Mansour, Fulvio Ferrara, Lucy Costantino, Carlo Setacci, Luca di Marzo

**Affiliations:** 1 Vascular and Endovascular Surgery Division, Department of General Surgery, Surgical Specialties & Anesthesiology, Policlinico Umberto I, Sapienza University of Rome, 00100 Rome, Italy; wassim.mansour@uniroma1.it (W.M.); luca.dimarzo@uniroma1.it (L.d.M.); 2 Integrated Laboratory Medicine Services, Centro Diagnostico Italiano, 20011 Milan, Italy; eugenio.caradonna@gmail.com; 3 Department of Experimental and Clinical Pathology, IRCCS Istituto Auxologico Italiano, 20149 Milan, Italy; f.ferrara@auxologico.it; 4 Laboratory of Medical Genetics, Centro Diagnostico Italiano S.p.A., 20011 Milan, Italy; lucy.costantino@cdi.it; 5 Vascular Surgery Department, Postgraduate School in Vascular Surgery, University of Siena, 53100 Siena, Italy; carlo.setacci@unisi.it

**Keywords:** TMAO, γ-butyrobetaine, oxidative stress, mitochondrial ROS, NLRP3 inflammasome, eNOS, SIRT3, gut microbiota, FMO3, peripheral artery disease

## Abstract

Background: Trimethylamine N-oxide (TMAO) arises from the interaction of diet, gut microbial metabolism, hepatic oxidation, and renal clearance. Experimental work links TMAO exposure to mitochondrial oxidative stress, NLRP3 inflammasome activation, impaired nitric oxide signaling, vascular smooth muscle cell dysfunction, and thrombosis. How far these findings explain human vascular disease remains uncertain. Purpose: We examine TMAO and related metabolites in carotid atherosclerosis, aortic disease (abdominal aortic aneurysm, AAA, and dissection), and peripheral artery disease (PAD), focusing on redox biology and the obstacles that still limit clinical translation. Position: Current evidence makes the pathway biologically credible, but it does not support routine TMAO measurement, a universal cutoff, or treatment decisions based on a single metabolite. The recent association between γ-butyrobetaine and limb outcomes also suggests that TMAO may not always be the most informative component of the pathway. Most causal evidence remains preclinical, and no TMAO-lowering or redox-directed intervention has improved a vascular clinical endpoint. Conclusions: For now, the TMAO pathway remains investigational. Progress will depend on multicenter studies that measure several pathway metabolites with harmonized assays and carefully account for renal function, diet, and sex. Interventional studies are premature until safety and biological target engagement have been established.

## 1. Introduction

Cardiovascular disease (CVD) remains the leading cause of death worldwide, with approximately 17.9 million deaths annually [1,2]. Population-level risk tools, such as the Framingham Risk Score, “SCORE2” and the American College of Cardiology/American Heart Association (ACC/AHA) Pooled Cohort Equations, draw on well-established epidemiological variables and perform reasonably well across populations. They perform far less well for individual patients in front of a vascular surgeon. None of them predicts which asymptomatic carotid plaque will destabilize, how fast a small abdominal aortic aneurysm (AAA) will outgrow its current diameter, or which patient undergoing revascularization for chronic limb-threatening ischemia (CLTI) will experience restenosis or lose the limb early. These are the decisions where a better biological readout would change our approach.

Interest in the gut microbiome as a cardiovascular contributor has grown steadily, particularly because microbial metabolites can influence host signaling. Among these metabolites, trimethylamine N-oxide (TMAO) has accumulated substantial evidence since the 2011 report by Wang et al., linking it to major adverse cardiovascular events [3]. Subsequent experimental work indicates that TMAO exposure can promote endothelial dysfunction, macrophage inflammatory signaling, vascular smooth muscular cell (VSMC) senescence, and platelet hyperreactivity in specific models. These findings do not establish that circulating TMAO is a causal driver in patients because plasma levels also integrate diet, microbial metabolism, hepatic flavin-containing monooxygenase-3 (FMO3) activity, and renal clearance. More recent data further complicate the model: γ-butyrobetaine, an intermediate of microbial L-carnitine catabolism, was associated with peripheral vascular outcomes independently of TMAO in one prospective cohort [4]. The clinically relevant readout may therefore be pathway- and endpoint-specific rather than TMAO alone.

Most mechanistic and prognostic work has focused on coronary artery disease (CAD), heart failure (HF), and chronic kidney disease (CKD). These studies have contributed substantially to the hypothesis that the TMAO pathway may reflect or participate in cardiovascular risk. However, the strength, independence, and clinical implications of these associations vary across populations and outcomes. The peripheral circulation—carotid, aortic, and lower-limb—has received comparatively less systematic attention, and no vascular-surgery guideline currently recommends routine TMAO measurement. A focused appraisal of the emerging territorial evidence is therefore warranted. The present paper therefore focuses specifically on carotid, aortic, and lower-limb arterial disease, where the translational relevance of the pathway remains less clearly defined.

Our purpose is to take a clear position on where the TMAO field stands in vascular medicine. We examine the mechanistic and clinical evidence across the main vascular territories, identify what is still missing for translation, and suggest a practical research agenda. The position is cautious: TMAO and its precursors are biologically credible candidates with prospective outcome associations, but the evidence does not support routine measurement, a universal threshold, or treatment decisions. Standardized prospective validation of the full metabolic axis—γ-butyrobetaine, TMA, and TMAO measured together under harmonized pre-analytical conditions—must come first.

### Scope and Basis of the Position

This is a position paper, not a systematic review or meta-analysis. We selected mechanistic, translational, and clinical studies that bear directly on carotid, aortic, and lower-limb disease. The aim is to explain the biological case, show where the evidence is weak or contradictory, and set out our priorities for research. We distinguish human observations from ex vivo, cellular, and animal experiments because they do not carry the same weight. The studies discussed were identified through non-systematic searches of PubMed and Scopus up to July 2026, supplemented by the reference lists of retrieved articles. Searches combined terms related to the TMAO metabolic pathway (including “trimethylamine N-oxide, “TMAO”, “trimethylamine”, TMA”, γ-butyrobetaine”) with terms referring to the vascular territories and diseases considered (“carotid”, “atherosclerosis”, “abdominal aortic aneurysm”, “aortic aneurysm”, “aortic dissection”, “peripheral artery disease”, and “critical/chronic limb-threatening ischemia”), together with mechanistic terms relevant to the scope of this paper, including “oxidative stress”, “reactive oxygen species”, “mitochondrial ROS”, “NLRP3”, “eNOS”, “SIRT3, and “vascular inflammation”. Priority was given to human studies directly addressing the vascular territories of interest, prospective studies reporting clinical outcomes, and experimental studies providing mechanistic evidence directly relevant to the TMAO-redox pathways discussed. Preclinical studies were included when they provided experimental guidance of the pathway or mechanistic information not available from human studies. Inclusion reflects relevance to the vascular territories considered rather than predefined eligibility criteria [5,6]. We explicitly distinguish human observational evidence from ex vivo, cellular, and animal experiments because these sources of evidence do not carry the same inferential weight. The positions set out below were drafted by the vascular surgical and laboratory medicine authors, circulated to all co-authors for comment, and revised iteratively until no author maintained a substantive objection; where interpretations diverged, the more conservative reading of the evidence was adopted. The recommendations that follow express the authors’ interpretation of the literature; they are not clinical practice guidance.

## 2. TMAO Pathophysiology: From Gut to Vascular Wall

**Figure 1:**
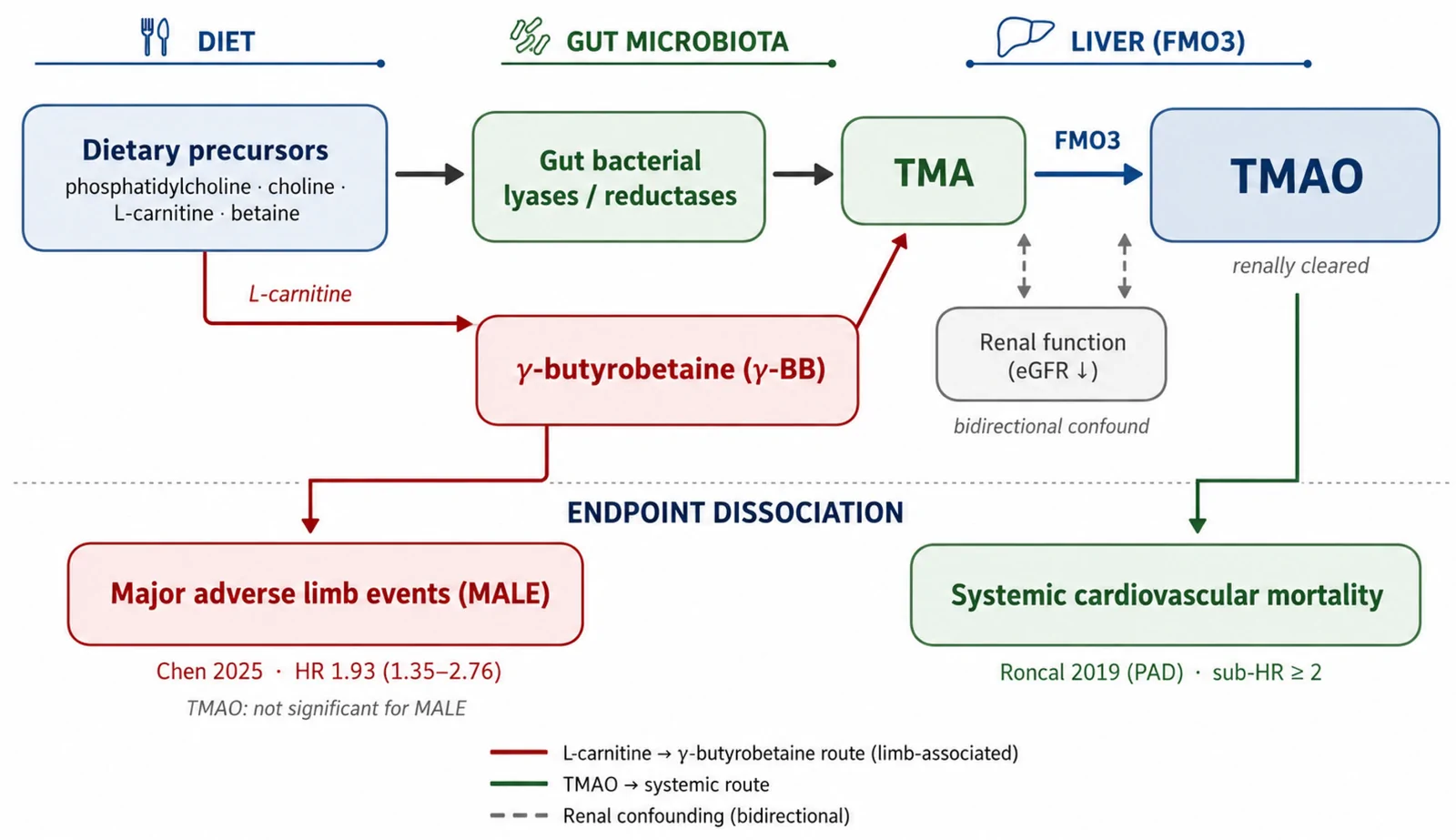
The TMAO metabolic axis and dissociation of vascular endpoints. Dietary quaternary amines are converted by gut bacteria to TMA and, along a microbial L-carnitine route, to the intermediate γ-butyrobetaine. Hepatic FMO3 oxidizes TMA to TMAO, which is cleared predominantly by the kidneys; renal function may act as a confounder, mediator, or time-varying covariate depending on the research question, depending on the estimated glomerular filtration rate (eGFR). Current prospective data suggest an endpoint split rather than a single culprit metabolite: TMAO was associated with systemic cardiovascular mortality [9], whereas γ-butyrobetaine, not TMAO, independently predicted major adverse limb events after lower-limb revascularization [4]. The solid arrows indicate the two routes; the dashed loop indicates the complex bidirectional relationship between circulating TMAO and renal function.

Three biological systems contribute sequentially to TMAO production [7]. Gut microorganisms convert dietary quaternary amines—including choline, L-carnitine, betaine, and phosphatidylcholine—into trimethylamine (TMA) through several taxon- and substrate-dependent pathways. In a major L-carnitine pathway, microbial oxidation generates γ-butyrobetaine (γ-BB) before onward conversion to TMA. γ-Butyrobetaine also circulates in plasma and has shown clinical associations that are not fully captured by TMAO [4], although independent pathogenicity remains to be established. TMA crosses the intestinal epithelium, reaches the liver through portal blood, and is oxidized predominantly by flavin-containing monooxigenase-3 (FMO3) to TMAO; TMAO is subsequently cleared mainly by the kidneys. A plasma TMAO value integrates recent dietary exposure, microbial metabolic capacity, hepatic oxidation, and renal handling (Figure 1). This composite origin complicates interpretation of a single measurement [8].

### 2.1. The Redox Core: TMAO, Mitochondrial ROS, and the Antioxidant Rationale

Across vascular territories, the experimental effects attributed to TMAO often converge on an imbalance between oxidant production and antioxidant defense. TMAO should not be exclusively considered a pathological metabolite. At physiological concentrations, TMAO functions as a chemical chaperone and osmolyte, maintaining protein folding and homeostasis under osmotic, thermal, and hydrostatic stress [10,11]. It stabilizes proteins by preferential exclusion from the protein surface, water structuring around the polypeptide backbone, and reinforcement of the hydration shell, thereby promoting native conformations [12]. Unlike glycine and betaine, TMAO stabilizes collapsed conformations through a unique surfactant-like mechanism acting on heterogeneous folded protein surfaces, not solely through preferential exclusion [13]. Among the common osmolytes, TMAO has the strongest protein-stabilizing effect and the highest osmotic coefficient [12]. TMAO also mitigates urea-induced protein denaturation by inhibiting preferential interactions between proteins and urea, a mechanism characterized in marine elasmobranchs and mammalian kidneys [14]. However, higher TMAO exposure has been associated with pro-oxidant and pro-inflammatory effects in experimental models [10].

In selected cell and mouse models, TMAO exposure amplifies oxidative and inflammatory signaling. One proposed mechanism begins with suppression of the mitochondrial deacetylase sirtuin 3 (SIRT3). The resulting increase in Superoxide Dismutase 2 (SOD2) acetylation reduces SOD2 activity and allows mitochondrial reactive oxygen species (mtROS) to accumulate [15]. Endothelial experiments link this signal to NOD-like receptor family pyrin domain-containing 3 (NLRP3) assembly, caspase-1 activation, and interleukin 1β (IL-1β) release [15]. Other reported effects include impaired endothelial nitric oxide synthase (eNOS)-dependent nitric oxide production and vasorelaxation [10], vascular smooth muscular cell (VSMC) senescence with induction of p16, p21, and matrix metalloproteinases [16], enhanced scavenger-receptor-mediated lipid uptake [3], and osteogenic VSMC programming associated with NLRP3 and nuclear factor kappa B (NF-κB) [17]. The convergence is persuasive, but it is not yet a unified human mechanism. In particular, the SIRT3–SOD2–mtROS pathway rests on endothelial-cell and ApoE-knockout mouse experiments [15], and its interpretation depends on dose, exposure time, cell type, and species. Because “reactive oxygen species” (ROS) is not a single endpoint, comparability across these studies is limited: reported readouts range from superoxide and hydrogen peroxide to lipid peroxidation and non-specific fluorescent probes, and the oxidant source (mitochondrial, NADPH-oxidase, or eNOS uncoupling) is frequently not resolved.

If oxidative stress (OS) contributes to TMAO-associated injury, the intervention must involve redox treatment, and trials of antioxidant vitamins in CVD have not reproduced the benefits seen in experimental models: in a meta-analysis of 50 randomized trials including 294,478 participants, vitamin and antioxidant supplementation was not associated with a reduction in major cardiovascular events (RR 1.00, 95% CI 0.98–1.02) [18]. The best developed upstream approach is inhibition of microbial TMA generation. Fluoromethylcholine and 3,3-dimethyl-1-butanol lowered TMAO and reduced vascular inflammation or aneurysm progression in mice [19,20]. FMO3 inhibition or knockdown also changes TMAO exposure, although methimazole is nonselective and cannot be regarded as a clinically usable FMO3 therapy. Mitochondria-targeted antioxidants, eNOS restoration, nuclear factor erythroid 2-related factor 2 (NRF2) modulation, and NLRP3 inhibition remain experimental in this setting. Taurisolo improved eNOS-dependent vasorelaxation in a preclinical model [10], but pharmacokinetic and target-engagement data are needed before that effect can be attributed to direct antioxidant activity. No antioxidant or TMA-lowering strategy has yet improved a vascular clinical endpoint. To distinguish practical barriers to translation from more fundamental uncertainties regarding human causality and clinical benefit, Table 1 summarizes the principal strategies targeting the TMAO metabolic and redox axis, their current level of evidence, major translational barriers, and the present research priority (Table 1).

### 2.2. Additional Priming Lesions: Leukocyte Adhesion, Thrombosis, and Impaired Repair

Beyond the redox core, TMAO primes the wall for leukocyte capture and thrombosis. Intercellular adhesion molecule 1 (ICAM-1), vascular cell adhesion molecule-1 (VCAM-1), and E-selectin increase through mitogen-activated protein kinase (MAPK) and NF-κB-dependent signaling, attracting leukocytes to the endothelium [21,22], while enhanced platelet collagen- and ADP-receptor expression tips the balance toward thrombosis [23]. TMAO also undermines repair; instable angina, higher plasma levels are associated with fewer and less functional circulating endothelial progenitor cells and worse endothelial function, implying a blunted capacity for compensatory angiogenesis [24].

A more speculative extension links TMAO with clonal hematopoiesis of indeterminate potential (CHIP). CHIP—clonal expansion of hematopoietic cells carrying somatic driver mutations, commonly in DNA methyltransferase 3 alpha (DNMT3A), ten-eleven translocation 2 (TET2), or additional sex combs like 1 (ASXL1)—is associated with inflammatory signaling. The CHIP–Dysbiosis–TMAO (CHIDT) hypothesis, previously advanced by some of the present authors, proposes a feed-forward interaction involving TET2 signaling [25]. Experimental observations cited in support of this framework include TMAO-associated inflammasome activation and proposed effects on endothelial TET2 and pyroptosis. However, the integrated loop has not been demonstrated prospectively in patients with vascular disease, and the physiological relevance of some experimental exposures remains uncertain. CHIP should therefore be presented as an exploratory effect modifier, not an established component of TMAO-mediated vascular injury [26].

### 2.3. TMAO, Metabolic Syndrome and Vascular Dysfunction

Metabolic syndrome provides an additional context in which the TMAO pathway may contribute to vascular dysfunction. Obesity, insulin resistance, dyslipidemia, and hypertension are associated with alterations in gut microbial metabolism and frequently coexist with renal and dietary factors that influence circulating TMAO. Higher TMAO concentrations have been reported in several cardiometabolic conditions, but these associations do not establish an independent causal role for TMAO. Mechanistically, experimental studies suggest that TMAO may aggravate endothelial dysfunction through increased ROS generation and inflammatory signaling, activation of NLRP3 and NF-κB pathways, increased expression of endothelial adhesion molecules, and impaired eNOS-dependent nitric oxide bioavailability. These mechanisms overlap substantially with those implicated in metabolic-syndrome-associated vascular injury and may therefore provide a biological interface between metabolic dysfunction and vascular disease. However, much of the mechanistic evidence derives from cellular and animal models, and the independent contribution of TMAO in patients with metabolic syndrome remains difficult to separate from obesity, diet, insulin resistance, renal function, and other conventional cardiovascular risk factors [27,28].

## 3. TMAO Across the Major Vascular Territories

### 3.1. Carotid Artery Disease

Carotid atherosclerosis accounts for approximately 20% of ischemic strokes [29,30]. Stratification still leans on three imaging-based descriptors: degree of stenosis, symptom status, and plaque morphology. The difficulty lies in asymptomatic disease—a large group whose absolute event risk on contemporary medical therapy has fallen considerably—where the case for intervention is often genuinely unclear, and a biological discriminator would earn its place.

Two independent lines of evidence converge in carotid disease, although both remain associative. The first is epidemiology. In vascular prevention clinics, plasma TMAO was higher in patients whose carotid total plaque area exceeded what their conventional risk factors predicted and lower in those with less plaque than expected, independent of renal function and dietary precursor intake [31]. In a separate cohort, TMAO tracked with carotid intima-media thickness (cIMT) after adjustment [32]. The second line is histopathology. Shi et al. [29] found that intraplaque FMO3 expression correlates with instability features, such as neovascularization, intraplaque hemorrhage, and fibrous-cap thinning, in endarterectomy specimens and that lowering TMAO experimentally shifted macrophages toward M2 polarization and efferocytosis. In redox terms, the carotid data point more to NLRP3/NF-κB-associated macrophage and VSMC programming than to the endothelial SIRT3–SOD2 branch, though the two need not be exclusive.

Sex deserves explicit attention, but the direction and mechanism of sex differences require careful interpretation. In the CORDIOPREV cohort (827 men and 175 women with coronary heart disease), García-Fernández et al. [30] reported higher TMAO levels and TMAO/TMA ratios in men, together with greater cIMT and plaque burden. This cohort-specific observation does not by itself establish generally higher intrinsic FMO3 activity in men, because diet, microbiota, renal clearance, medication, age, and hormonal status can also influence circulating TMAO and the TMAO/TMA ratio. Foundational expression data reported lower hepatic FMO3 expression in males than in females, in both humans and mice [33]; the sex effect is therefore context-dependent rather than a fixed male-versus-female difference. Sex and hormonal status should therefore be prespecified covariates, and mechanistic inference should not be drawn from a metabolite ratio alone. Experimental reduction of TMAO through FMO3-directed approaches has affected plaque phenotypes in mice [34], but methimazole is nonselective and no human pharmacological evidence supports FMO3 as a current therapeutic target [35].

### 3.2. Aortic Disease: Aneurysm and Dissection

Abdominal aortic aneurysm (AAA) is found in roughly 1.5–2.0% of men over 65 [36]. Surveillance rests almost entirely on one anatomical number, aortic diameter, which loosely maps onto rupture risk: some aneurysms rupture below the operative threshold, while others remain quiescent for years. What is missing is a size-independent readout of the inflammatory and proteolytic activity inside the wall—the kind of marker that could sharpen the timing of intervention.

In AAA the evidence runs deeper than anywhere else in the peripheral circulation, although most of that depth is preclinical. Several datasets converge in a mechanistic-to-clinical sequence, and it is worth keeping track of which links are human and which are murine. Mechanistically, in two independent U.S. and European cohorts (n = 2129), Benson et al. [16] found that elevated TMAO levels were associated with AAA incidence and growth rate. In murine models, dietary choline supplementation augmented aortic diameter in two independent AAA models (AngII infusion and elastase); this effect was suppressed by broad-spectrum antibiotics and abrogated by targeted inhibition of microbial TMA lyase (CutC/D) with fluoromethylcholine, an approach that halted the progression of pre-existing AAA in mice. FMO3-knockout mice were protected from AAA rupture, and RNA sequencing identified the protein kinase R (PKR)-like endoplasmic reticulum kinase (PERK) as a key downstream mediator [37].

A second murine study [13] added VSMC senescence to the list, with TMAO raising p21, p16, ROS, Matrix Metalloproteinase-2 (MMP-2), and Matrix Metalloproteinase-9 (MMP-9) in AngII- and CaCl_2_-induced models. Wei et al. [17] carried the work into human tissue: plasma TMAO was elevated in AAA patients relative to non-aneurysmal controls, and the operative mechanisms were NF-κB-mediated M1 macrophage polarization and VSMC phenotypic switching, both suppressed by the TMA-lyase inhibitor 3,3-dimethyl-1-butanol (DMB).

The AAA evidence therefore spans endoplasmic reticulum (ER) stress signaling, VSMC senescence, and NF-κB-associated inflammatory switching. These mechanisms converge conceptually on wall degeneration, but most causal evidence is derived from mouse models and cannot yet define a therapeutic target in patients [38]. CutC/D-inhibitor experiments provide proof of mechanism in animals; translation will require a clinical-grade compound, toxicology, pharmacokinetics, target-engagement biomarkers, and staged safety testing [39]. Among the human datasets, the multicenter findings reported by Cameron et al. [33], together with a large community-based cohort (Li et al. [35]: 4442 older adults in the Cardiovascular Health Study followed for a median of 12 years; adjusted HR 1.28 per doubling of TMAO and 2.46 for the top versus bottom tertile of TMAO, for adverse AAA events), strengthen the prognostic association and the rationale for prospective validation. They do not, however, establish causality, a treatment threshold, incremental clinical utility, or a biomarker-guided surveillance strategy. Mechanistically, the aortic signal is dominated by ER stress and ROS-driven VSMC senescence rather than by the endothelial redox branch.

Thoracic aortic aneurysm (TAA) should be considered separately from AAA because the two conditions differ substantially in anatomical distribution, genetic background, extracellular matrix biology, and clinical determinants. In contrast to the growing evidence linking the TMAO pathway to AAA, direct evidence for a role of circulating TMAO in the development or progression of human TAA remains limited. Although mechanisms implicated in TMAO-associated vascular injury—including oxidative stress, endothelial dysfunction, inflammatory signaling, VSMC phenotypic alterations, and extracellular matrix remodeling—are biologically relevant to thoracic aortic degeneration, their involvement does not establish a TMAO-specific mechanism in TAA. Current evidence therefore supports TMAO as a plausible modifier rather than an established biomarker or causal mediator of TAA. Dedicated prospective cohorts measuring TMAO and related pathway metabolites alongside thoracic aortic growth, genotype, aortic phenotype, and clinical outcomes are needed before the findings obtained in AAA can be extrapolated to thoracic aneurysmal disease [5].

For aortic dissection the evidence base has shifted, and the earlier premise that no clinical data existed no longer holds. Two case-control studies have now quantified circulating TMAO in dissection patients. Zeng et al. [37] found elevated serum TMAO in nineteen patients with Stanford type A dissection relative to controls, with TMAO correlating with C-reactive protein, interleukin-6 (IL-6), D-dimer, and the maximum aortic diameter recorded on admission. In a larger series, Huang et al. [38] measured markedly higher plasma TMAO in 253 dissection patients than in 98 healthy subjects (median 3.47 vs. 1.85 µmol/L, *p* < 0.001), with higher values tracking disease severity and with 16S sequencing showing enrichment of TMA-producing genera; the same group reported that a TMAO-supplemented diet promoted dissection in AngII- and β-aminopropionitrile (BAPN)-induced rodent models, whereas TMAO depletion attenuated it, implicating endothelial dysfunction and NF-κB activation. In evidentiary shape, dissection now resembles AAA—human association plus murine causal manipulation—rather than an untested territory. The caveats are familiar but particularly acute here: the human series are cross-sectional and cannot separate cause from consequence in a catastrophic event, where TMAO may rise secondarily to renal hypoperfusion, malperfusion, or the acute-phase response; recent syntheses still classify the gut–dissection link as a plausible modifier rather than an established driver [40]. No prospective or interventional human data exist.

### 3.3. Peripheral Artery Disease

An estimated 200–300 million people worldwide live with peripheral artery disease (PAD) [41]. At the severe end of the spectrum, chronic limb-threatening ischemia (CLTI) shows stark numbers: approximately 30% major amputation rate and 20–25% mortality within a year of diagnosis. Outcomes after revascularization vary widely among patients, and no preoperative biomarker has yet been identified that reliably stratifies this risk [42].

In PAD the mechanistic rationale mirrors that of the other territories and is not restated here. Which redox node dominates in the limb is not yet defined, and the γ-butyrobetaine signal noted below may be only loosely coupled to it. What matters clinically is the host. Patients with atherosclerotic vascular disease carry a dysbiotic signature characterized by enrichment of pro-atherogenic, TMA-producing taxa and depletion of short-chain fatty acid producers, and this profile is generally assumed to extend to PAD [6]. The assumption has not been tested: we identified no cohort study that specifically characterized the fecal microbiome in a PAD population, and the taxa described come from coronary, cerebrovascular, and mixed vascular cohorts. In addition, a high prevalence of chronic kidney disease raises circulating TMAO levels through reduced clearance alone [40]. The two effects are hard to separate at the bedside.

The main prospective signal comes from Roncal et al. [9], who tracked 262 patients with symptomatic PAD for a mean of four years. A plasma TMAO level above 2.26 µmol/L, a receiver operating characteristic (ROC)-derived cutoff with 62% sensitivity and 76% specificity, independently predicted cardiovascular mortality (sub-hazard ratio ≥ 2, *p* < 0.05) once traditional risk factors, including estimated glomerular filtration rate (eGFR), were considered. Levels were higher in critical limb ischemia than in claudication, and the mortality association survived eGFR adjustment; even so, the inverse TMAO–eGFR correlation was a reminder that renal function must be controlled for deliberately and not assumed away.

A 2025 prospective study introduced an important refinement to the TMAO–PAD framework. Chen et al. [4] prospectively enrolled 395 patients with symptomatic PAD (mean age 72.2 years, 61% male, follow-up 1.5 years) undergoing endovascular therapy, with major adverse limb events (MALEs) (revascularization and amputation) as the primary outcome. γ-Butyrobetaine, an intermediate in microbial L-carnitine catabolism upstream of TMA and TMAO, independently predicted MALE with a hazard ratio (HR) of 1.93 (95% CI 1.35–2.76) after multivariable adjustment. TMAO showed no significant association with MALE risk, although both metabolites were associated with major adverse cardiovascular events (MACE). This pattern suggests that the axis operates differentially, with TMAO associated with systemic cardiovascular mortality and γ-butyrobetaine associated with the limb outcome in this cohort. This finding should be considered hypothesis-generating and requires validation in independent, adequately powered PAD cohorts before any conclusion can be drawn regarding the relative prognostic performance of these metabolites. Moreover, whether this reflects a distinct pro-thrombotic or vasoconstrictive action on the peripheral arterial bed is an untested hypothesis, and the dissociation itself requires external replication before it can be treated as a biological rule. If the dissociation holds, it is an uncomfortable result for a field that has spent more than a decade treating TMAO as the molecule of interest. For the one outcome a vascular surgeon most wants to predict—whether the limb survives—it was the precursor, not the end product, that carried the signal. This possibility deserves to be tested directly rather than being assumed. Any prospective PAD study should therefore measure γ-butyrobetaine alongside TMAO and TMA, so that the analysis can ask directly which member of the axis best predicts MALE, patency, and CLTI-specific outcomes after revascularization. Accordingly, concurrent measurement of γ-butyrobetaine, TMA, and TMAO may be more informative for investigating the metabolic axis than measurement of TMAO alone, because it allows their independent and incremental prognostic contributions to be tested within the same population. Whether such multi-metabolite profiling improves clinical risk prediction remains to be demonstrated prospectively. Dietary and microbiome-directed modulation is more feasible in PAD than in carotid or aortic disease. A hypocaloric diet plus exercise lowers TMAO by approximately 31% compared with eucaloric exercise alone in obese adults [43], although no vascular-specific clinical benefit has been shown (Table 2).

## 4. Current Limitations Before Clinical Translation

The evidence assembled in Section 2 and Section 3 is mechanistically coherent and clinically suggestive; however, suggestive is not the same as sufficient, and none of it warrants routine TMAO measurement in vascular surgical practice. Several distinct problems stand in the way, and each must be dealt with before clinical use could be justified.

### 4.1. Absence of Standardized Measurement

TMAO is commonly measured by liquid chromatography–tandem mass spectrometry, preferably with stable-isotope dilution [44]. Specialized laboratories can perform the assay, but protocols differ in sample handling, calibration, internal standards, and reporting units. There are also no universally accepted clinical decision thresholds. γ-Butyrobetaine presents the same problem: Chen et al. used HPLC-MS/MS [4], but the assay has not undergone external cross-laboratory validation. The obstacle is therefore harmonization and clinical validation, not simply access to mass spectrometry.

### 4.2. Dietary Confounding

Plasma TMAO depends strongly on what and when a person has eaten. Fish contains preformed TMAO and can raise circulating concentrations without microbial conversion. Red meat, eggs, and other precursor-containing foods produce more variable responses because microbial metabolism and renal handling also matter [45]. In a controlled crossover study, the acute increase after fish was substantially greater than after eggs or beef. This is difficult to reconcile with the cardiovascular benefits associated with many fish-rich diets and illustrates why a single TMAO value can mislead. Vascular studies need a record of recent food intake and habitual diet, a defined fasting interval, and preferably repeated samples. A 72 h low-choline washout or a target below 300 mg/day cannot be recommended without evidence that it is valid, feasible, and nutritionally acceptable [45].

### 4.3. The TMAO–Renal Function Relationship: Confounder, Mediator, or Time-Varying Covariate

TMAO is cleared by the kidneys; therefore, its plasma concentration increases as glomerular filtration decreases. The community-based cohort of Wang et al. [44]—10,564 adults with serial measurements—showed the relationship runs the other way too: higher TMAO independently predicted incident CKD (HR 2.24, 95% CI 1.68–2.98, fifth versus first quintile) and faster eGFR decline (−0.43 mL/min/1.73 m^2^ per year), with dose–response relationship holding across racial and ethnic groups. Renal function therefore cannot be treated as a single kind of variable: depending on the question asked, it may act as a confounder, as a mediator on the causal path between TMAO exposure and vascular events, or as a time-varying covariate when serial measurements are available. The possibility that TMAO itself hastens renal injury, perhaps through tubulointerstitial toxicity, rests on experimental and observational data and should be presented as a hypothesis rather than an established mechanism. In PAD and AAA, where CKD is common, the analytical role assigned to eGFR should be prespecified, since routine adjustment biases the estimate when renal function lies on the causal path; no adjustment strategy has yet been validated in vascular cohorts [40].

### 4.4. Metabolite Specificity: TMAO Versus γ-Butyrobetaine

Chen et al. [4] raise a question the field can no longer avoid: which component of the L-carnitine–γ-butyrobetaine–TMA–TMAO pathway predicts which outcome? TMAO has been associated with systemic outcomes, whereas γ-butyrobetaine carried the limb signal in this cohort. That pattern may be real, but it rests on limited data and needs replication. Future studies would be more informative if they measured several pathway metabolites together and tested whether any of them adds prognostic information beyond renal function, diet, conventional risk factors, and the other metabolites.

### 4.5. Absence of Validated Clinical Thresholds

More than a decade of association studies has not produced a single TMAO threshold with validated sensitivity, specificity, predictive value, and likelihood ratios for any vascular surgical decision, including carotid risk stratification, AAA surveillance intensity, and peri-procedural risk in PAD. The 2.26 µmol/L cutoff proposed by Roncal et al. [9] to separate critical limb ischemia from claudication has not been externally validated in an independent prospective cohort, nor outside a Mediterranean population. Without a validated cutoff there can be no decision support.

### 4.6. Absence of Prospective Interventional Evidence

All current clinical evidence in carotid, aortic, and lower-limb disease is observational; no randomized trial has shown that modifying TMAO or a downstream redox pathway improves a vascular clinical outcome. Association does not establish clinical utility. In 1726 patients with acute ischemic stroke in the multicenter, predominantly White BIOSIGNAL cohort, TMAO was not independently associated with recurrent stroke (adjusted HR 1.07, 95% CI 0.78–1.47) or MACE (adjusted HR 0.90, 95% CI 0.74–1.09), although a modest association with poor functional outcome remained (adjusted OR 1.28, 95% CI 1.04–1.57) [46]. The investigators interpreted elevated TMAO primarily as a marker integrating renal dysfunction and cardiovascular risk burden. These findings emphasize population heterogeneity and the need for external validation rather than disproving all experimental mechanisms.

### 4.7. Biological and Pharmacological Confounders

FMO3 expression and circulating TMAO vary with sex, hormonal status, age, diet, genetics, renal function, and possibly sampling time. The sex-stratified literature is not uniform. Men in CORDIOPREV had a higher TMAO/TMA ratio [47], whereas earlier studies found lower hepatic FMO3 expression in males [33]. Medication is another unresolved source of variation: antibiotics, metformin, proton pump inhibitors, and other microbiome-modifying drugs may alter pathway metabolites, but their effects have not been mapped systematically in vascular cohorts. The proposed interaction between TMAO and CHIP also remains a hypothesis in the absence of direct human evidence.

### 4.8. Potential Roles Before Clinical Decision Support

The absence of sufficient evidence for TMAO-guided clinical decisions does not preclude more limited roles for TMAO and related pathway metabolites in vascular research. Before reaching the threshold required for routine clinical decision support, these metabolites could be evaluated as tools for risk stratification or cohort enrichment, particularly if they provide prognostic information beyond established clinical, anatomical, and renal variables. Multi-metabolite profiling may also support mechanistic phenotyping by identifying patient subgroups with different patterns of pathway activation and may help clarify whether TMAO, TMA, γ-butyrobetaine, or their combination is most closely associated with specific vascular outcomes. In early-phase intervention studies, changes in these metabolites could additionally serve as pharmacodynamic or target-engagement biomarkers, providing evidence that a dietary, microbiome-directed, or pharmacological intervention has modified the intended metabolic pathway [43]. These applications require a lower level of validation than using a biomarker to direct treatment, but they still require standardized assays, control of major confounders, and prospective validation. Importantly, a pharmacodynamic response should not be equated with surrogate-endpoint validity. Demonstrating that an intervention lowers circulating TMAO would establish biochemical pathway modulation, but not that the observed change predicts clinical benefit. Validation as a surrogate endpoint would require evidence that treatment-induced changes in the biomarker reliably reflect corresponding changes in clinically meaningful vascular outcomes. Such evidence is currently lacking for TMAO and related metabolites in carotid, aortic, and lower-limb arterial disease [7].

## 5. Position on Research Priorities and Proposed Study Frameworks

We outline three multicenter study concepts that could test the position developed in this paper. They are not ready-made protocols. Each would need feasibility work, patient input, formal statistical planning, and adaptation to the participating networks. What they share is more important than their individual details: central measurement of γ-butyrobetaine, TMA, and TMAO using the same pre-analytical and analytical procedures. Each should also carry a small, harmonized redox panel measured centrally rather than a generic index of total antioxidant capacity, so that the pathway’s redox rationale is tested rather than assumed. A workable minimum would be F2-isoprostanes for lipid peroxidation, protein carbonyls or the oxidized-to-reduced glutathione ratio for protein and thiol redox status, and 3-nitrotyrosine for nitrosative damage. For each analyte the protocol must specify the biological matrix, the anticoagulant, the interval from venepuncture to processing, the antioxidant additives required to prevent ex vivo oxidation, the storage temperature, the permitted number of freeze–thaw cycles, and an acceptable inter-laboratory coefficient of variation established on shared quality-control samples before enrolment opens.

### 5.1. TMAO-CAROTID Study

The TMAO-CAROTID concept is a prospective cohort of patients with ≥50% carotid stenosis, with a separately powered dietary-feasibility substudy. A central laboratory would measure fasting TMAO, γ-butyrobetaine, TMA, and choline using validated stable isotope dilution LC-MS/MS. Diet, medication exposure, eGFR, and albuminuria would be recorded at sampling, and repeat measurements could quantify within-person variation. Symptomatic and asymptomatic patients should be enrolled in prespecified strata, since baseline event rates, treatment pathways, and the clinical question a biomarker would answer differ between the two groups. The primary clinical endpoint would be ipsilateral ischemic stroke, with non-stroke death handled as a competing risk rather than as censoring; TIA is better analyzed separately because its diagnosis is less secure. Biomarkers should be modeled continuously, allowing for nonlinear associations, instead of being reduced to data-derived quartiles. Plaque progression, post-revascularization events, intraplaque FMO3, and CHIP may be informative secondary or exploratory measures. The event count is the practical constraint. At a 2% annual stroke rate, 1200 participants would produce only about 48 events over two years before attrition, too few for the proposed stratified models. We present this figure as a feasibility check rather than a power calculation; event-based statistical sizing would be required before the design could proceed.

### 5.2. TMAO-AORTA Study

The TMAO-AORTA concept would sit within existing AAA surveillance programs. Patients with small aneurysms would undergo central measurement of TMAO, γ-butyrobetaine, and TMA at enrollment and follow-up, together with renal, dietary, medication, and imaging data. Rather than calculating a simple change between two scans, the primary analysis would use all available diameter measurements from a single prespecified imaging modality (ultrasound or computed tomography (CT), but not a mixture of the two), acquired to a common protocol and read centrally by an imaging core laboratory blinded to metabolite concentrations; model residual intra- and inter-observer variability explicitly; and account for measurement error, repair, and death. Repair threshold, rupture, aneurysm-related death, and all-cause mortality are distinct outcomes and need separate analyses. CHIP could be explored if the cohort is large enough. We would not embed a first-in-patient CutC/D inhibitor trial at this stage. That work belongs in a separate development pathway beginning with a clinical-grade compound, toxicology, pharmacokinetics, and target-engagement studies. Cohort size will depend on the number and timing of scans, within-person correlation, attrition, and the smallest growth difference considered clinically important.

### 5.3. TMAO-PAD Study

For PAD, we envisage a cohort of patients undergoing lower-limb revascularization for chronic limb-threatening ischemia or severe claudication, paired with a separate randomized dietary-feasibility substudy. Disease severity would be captured with a contemporary limb-staging system as well as the Rutherford category. A central assay would measure TMAO, γ-butyrobetaine, TMA, L-carnitine, choline, and betaine before treatment and during follow-up. The primary endpoint would be a prespecified major adverse limb event (MALE) composite—defined a priori from reintervention, thrombosis, and major amputation, with vascular death handled as a competing risk rather than folded into the composite—and its individual components would also be reported. All candidate events would be adjudicated by an independent committee blinded to metabolite concentrations, working from prespecified definitions. TMAO and γ-butyrobetaine can both be primary biomarkers only if the analysis accounts for multiplicity and has adequate power. Patency, ankle-brachial index, wound healing, mortality, and MACE are reasonable secondary outcomes if definitions and adjudication are standardized. The dietary substudy should define the intervention explicitly—the targeted reduction in dietary quaternary-amine precursors, its mode of delivery (dietitian-led counselling, provided meals, or both), and its duration—against a stated comparator such as standard dietary advice, and should verify adherence with objective measures rather than self-report alone: food records validated against 24 h urinary excretion of TMAO and carnitine, session attendance, and the expected fall in plasma pathway metabolites. Its first objectives are adherence, nutritional adequacy, biomarker response, and safety. It requires its own sample size calculation and analysis plan, separate from the parent cohort.

## 6. Conclusions

TMAO and its precursors form a gut-derived metabolic pathway with plausible links to vascular injury. The evidence is not uniform across carotid, aortic, and lower-limb disease, and most of it remains preclinical or observational. Two recent findings sharpen the question rather than settle it: the identification of VSMC senescence and microbial TMA-lyase-dependent mechanisms in AAA [16,19,20], and the prospective association of γ-butyrobetaine, rather than TMAO, with major adverse limb events in PAD [4]. Together, these findings support a multi-metabolite strategy, but they do not establish human causality or clinical utility.

Whether TMAO is associated with vascular disease is no longer the most interesting question. What remains unclear is which metabolite predicts which outcome, in whom, and how much of the apparent signal reflects renal function. Repeated measurements may prove more useful than a single concentration. Any intervention must first show that it safely engages the intended biological target.

The studies outlined in Section 5 are research priorities, not finished protocols. Their common features are central multi-metabolite assays, repeated assessment of renal function and diet, sex-aware analyses, and consistent outcome definitions. Feasibility and statistical design will determine what can be delivered. CHIP is best kept exploratory until a direct interaction with the TMAO pathway is demonstrated in patients.

The underlying hypothesis is simple enough to test: a diet-sensitive, microbiome-derived pathway may shape the course of vascular disease. Whether it matters at the bedside remains an open question. TMAO and related metabolites will need to improve prediction beyond established models, and modifying the pathway will need to benefit patients, not merely lower a laboratory value. Until then, they remain research biomarkers rather than clinical decision tools.

## Figures and Tables

**Table 1:** Translational feasibility of strategies targeting the TMAO metabolic and redox axis.

Strategy	Rationale	Current Evidence	Main Barrier	Barrier Type	Translational Priority
**Dietary modification**	Reduce precursor availability	Human TMAO lowering; no vascular outcome evidence	Adherence, nutritional effects, uncertain causal benefit	Technical + biological	Feasibility testing
**Microbial TMA-lyase inhibition**	Reduce TMA upstream	Strong animal proof-of-mechanism	Clinical-grade compounds, toxicology, PK, microbiome effects	Mainly technical, but clinical principle unproven	Promising preclinical
**FMO3 modulation**	Reduce TMAO conversion	Genetic/pharmacological experimental evidence	Non-selectively, systemic consequences of FMO3 manipulation	Technical + principle/safety	Caution
**Microbiome modulation**	Alter TMA-producing capacity	Biologically plausible, heterogeneous	Specificity, durability, interindividual microbiome variation	Technical + biological	Exploratory
**Downstream redox targeting**	mtROS/nLRP3/eNOS/Nrf2	Preclinical	Pathway specificity and target engagement	Principle + technical	Mechanistic studies
**Conventional antioxidants**	Reduce oxidative injury nonspecifically	Cardiovascular trials largely negative	Lack of pathway specificity	Principle	Low priority as nonspecific strategy
**Natural compounds**	Putative antioxidant/TMAO effects	Mostly preclinical	Composition, PK, target engagement, mechanism	Technical + evidentiary	Exploratory

**Table 2:** Principal clinical studies of the TMAO metabolic axis in major vascular territories. The rows are grouped by vascular territory and, within each territory, ordered by study design. Abbreviations: cIMT, carotid intima-media thickness; γ-BB, γ-butyrobetaine; MALE, major adverse limb events; MACE, major adverse cardiovascular events; sub-HR, subdistribution hazard ratio; CHD, coronary heart disease; PAD, peripheral artery disease; AAA, abdominal aortic aneurysm; FMO3, flaving-monooxigenase-3; AngII, angiotensin II; CRP, C-reactive protein; BAPN, β-aminopropionitrile; eGFR, estimated glomerular filtration rate. The arrow ↓ means decrease, the arrow ↑, means increase. Note the endpoint dissociation in PAD and the null result in a predominantly White stroke cohort (BIOSIGNAL), included here to maintain the balance of evidence.

Vascular Territory	Principal Study	Design	Population	Metabolite(s) Measured	Endpoint	Main Result	Main Limitation
**Carotid**	Bogiatzi 2018	Case-referent (prevention clinic)	Extremes of carotid plaque area	TMAO (+dietary precursors)	Total carotid plaque area	TMAO higher than predicted in the high-plaque extreme, independent of renal function and diet	Cross-sectional; surrogate endpoint; no clinical events
**Carotid**	García-Fernández 2025 (CORDIOPREV)	Prospective cohort, sex-stratified	827 M/175 F with CHD	TMAO, TMA (TMAO/TMA ratio)	cIMT; carotid plaque burden	Men had higher TMAO and TMAO/TMA ratios in this CHD cohort, with greater cIMT and plaque burden; the metabolite ratio does not establish generally higher intrinsic FMO3 activity	Coronary (not surgical) cohort; carotid is a secondary readout
**AAA**	Benson 2023	Human cohorts + murine (AngII, elastase)	N = 2129 human + mouse models	TMAO, choline	AAA incidence and growth rate	Higher TMAO was associated with AAA incidence and growth in human cohorts; targeted microbial TMA-lyase inhibition attenuated progression in mouse models	Intervention evidence murine only; not yet tested in patients
**AAA**	Cameron 2025	Multicenter prospective (clinical)	AAA surveillance patients	TMAO	Growth rate; surgical risk	Circulating TMAO was associated with aneurysm growth rate and surgical risk	Single metabolite (no γ-BB/TMA); thresholds unvalidated
**AAA**	Li 2026	Prospective cohort study	4442 adults ≥ 65 y	TMAO	Adverse AAA events (repair/rupture/death)	TMAO associated with adverse AAA events: Hazard Ratio 1.28 per doubling; 2.46 (T3 vs. T1)	Observational; no causality, threshold, or incremental utility
**Aortic dissection**	Zeng 2020	Case-control metabolomics	19 Stanford type A vs. 20 controls	TMAO (+precursors)	Diagnostic biomarker	TMAO elevated; correlates with CRP, Interleukin-6, D-dimer, max aortic diameter	Very small n; cross-sectional
**Aortic dissection**	Huang 2024	Case-control + murine (AngII, BAPN)	253 AD vs. 98 controls	TMAO	Aortic dissection severity	TMAO higher in AD (3.47 vs. 1.85 µmol/L); TMAO diet ↑, depletion ↓ dissection in mice	Cross-sectional human data; reverse causation in acute event
**PAD**	Roncal 2019	Prospective cohort (~4 y)	262 symptomatic PAD	TMAO	Cardiovascular mortality	TMAO > 2.26 µmol/L predicts cardiovascular mortality (sub-HR ≥ 2), eGFR-adjusted	ROC-derived cutoff not externally validated; renal confounding
**PAD**	Chen 2025	Prospective cohort, endovascular (1.5 y)	395 symptomatic PAD	TMAO, γ-butyrobetaine, TMA	MALE (primary); MACE	γ-butyrobetaine predicts MALE (HR 1.93); TMAO not significant for MALE	Short follow-up; single-center assay; γ-BB not standardized
**Cerebrovascular (stroke)**	Frenger 2026 (BIOSIGNAL)	Multicenter prospective	1726 acute ischemic stroke (mostly White)	TMAO	Recurrent stroke; MACE	TMAO not independently associated after adjustment (recurrent stroke aHR 1.07; MACE aHR 0.90)	Likely renal/risk-factor confounding; population-specific

## Data Availability

No new data were created or analyzed in this position paper. Data sharing is therefore not applicable.

## Abbreviations

**TMAO**	Trimethylamine N-oxide
**CVD**	Cardiovascular disease
**ACC/AHA**	American College of Cardiology/American Heart Association
**AAA**	Abdominal aortic aneurysm
**CLTI**	Chronic limb-threatening ischemia
**VSMC**	Vascular smooth muscular cells
**FMO3**	Flavin-containing monooxygenase-3
**CAD**	Coronary artery disease
**HF**	Heart failure
**CKD**	Chronic kidney disease
**TMA**	Trimethylamine
**γ-BB**	γ-butyrobetaine
**SIRT3**	Sirtuin 3
**NLRP3**	NOD-like receptor family pyrin domain-containing 3
**IL-1β**	Interleukin 1β
**eNOS**	Endothelial nitric oxide
**NF-κB**	Nuclear Factor kappa B
**ROS**	Reactive oxygen species
**OS**	Oxidative stress
**NRF2**	Nuclear factor erythroid 2-related factor 2
**ICAM-1**	Intercellular adhesion molecule 1
**VCAM-1**	Vascular cell adhesion molecule-1
**MAPK**	Mitogen-activated protein kinase
**DNMT3A**	DNA methyltransferase 3 alpha
**TET2**	Ten-eleven translocation 2
**ASXL1**	Additional sex combs like 1
**CHIDT**	CHIP-dysbiosis-TMAO
**cIMT**	Carotid intima-media thickness
**PERK**	Protein kinase R-like endoplasmic reticulum kinase
**MMP-2**	Matrix metalloproteinase-2
**MMP-9**	Matrix metalloproteinase-9
**DMB**	3,3-dimethyl-1-butanol
**ER**	Endoplasmic reticulum
**TAA**	Thoracic aortic aneurysm
**IL-6**	Interleukin-6
**BAPN**	β-aminopropionitrile
**PAD**	Peripheral artery disease
**ROC**	Receiver operating characteristics
**eGFR**	Estimated glomerular filtration rate
**MALE**	Major adverse limb events
**HR**	Hazard ratio
**MACE**	Major adverse cardiovascular events
**CHIP**	Clonal hematopoiesis ok indeterminate potential
**CT**	Computed tomography
